# Relationship between transition shock, psychological contract, and resilience among new graduate nurses: a latent profile analysis

**DOI:** 10.3389/fpubh.2026.1834039

**Published:** 2026-06-09

**Authors:** Daijun Chen, Zhun Li, Li Wang, Adan Fu, Xi Xia, Tian Tian, Weilai Zhou, Yuqin Hu, Ting Chen

**Affiliations:** 1 Department of Gastroenterology, The Central Hospital of Wuhan, Tongji Medical College, Huazhong University of Science and Technology, Wuhan, Hubei, China; 2 Key Laboratory for Molecular Diagnosis of Hubei Province, The Central Hospital of Wuhan, Tongji Medical College, Huazhong University of Science and Technology, Hubei, China; 3 Department of Nursing, The Central Hospital of Wuhan, Tongji Medical College, Huazhong University of Science and Technology, Wuhan, Hubei, China

**Keywords:** latent profile analysis, new graduate nurses, psychological contract, resilience, transition shock

## Abstract

**Aim:**

As the healthcare industry evolves, new graduate nurses encounter challenges transitioning from students to practitioners, impacting team stability and service quality. Many experience transition shock, marked by role confusion, skill gaps, and interpersonal pressures. Understanding the factors contributing to this phenomenon is crucial. However, recent studies have primarily focused on the overall level and influencing factors of transition shock, overlooking the heterogeneity within this population. This study aims to assess the current state of transition shock among new graduate nurses and explore its influencing factors.

**Methods:**

A convenience sampling, cross-sectional survey was conducted with 220 new graduate nurses from September 2024 to October 2024 in a tertiary hospital in China. Participants were administered by general condition questionnaire, the Transition Shock Scale, the Connor-Davidson Resilience Scale, and the Nurse Psychological Contract Scale. A latent profile analysis was used to identify the latent profiles of transition shock. Univariate and multivariate logistic regression analyses were used to explore the predictors of different profiles.

**Results:**

The transition shock of new graduate nurses could be classified into three profiles: low transition shock, middle transition shock, and high transition shock. Job satisfaction, personality, resilience, and psychological contract were predictors of different profiles.

**Conclusion:**

In this study, the majority of new graduate nurses were classified into Class 2 and Class 3, and their transition shock was relatively high. Nursing managers ought to focus on reducing transition shock for new graduate nurses. Key factors like personality, job satisfaction, resilience, and psychological contract should guide the creation of specific management actions.

## 1 Introduction

The shortage of nursing personnel has become a global issue affecting numerous countries (1). The high turnover rate among new graduate nurses has exacerbated this shortage, further contributing to the deficit in nursing staff (2). A longitudinal study found that 71.8% of new graduate registered nurses intended to resign during their first year on the job (3). Research indicates that a primary factor driving turnover among these new graduates is the substantial transition shock they experience (4). This phenomenon can adversely impact their physical and psychological wellbeing, potentially leading to work adjustment disorders and increasing the likelihood of nursing-related adverse events, thereby posing a threat to the quality of care and patient safety (5, 6).

According to Duchscher’s transition shock theory, transition shock refers to the frequent experiences of confusion and uncertainty encountered by new graduated nurses as they transition from nursing students to professional practitioners (7). This transition requires significant adjustments in their roles, work environment, tasks, responsibilities, and interpersonal dynamics. Recognizing the factors that contribute to transition shock can guide the creation of support strategies to make transitions smoother and better integrate new nurses into the workforce (33–35). Given the importance of transition shock among new graduate nurses, it is crucial to identify potential profiles of new graduate nurses’ transition shock and further explore the predictors of different profiles to develop effective strategies to decrease nurses’ transition shock. Despite previous studies identifying factors contributors to transition shock (8), few studies have investigated this issue from a latent profile perspective.

Resilience is defined as an individual’s capacity to recover and adapt in the face of adverse events, including adversity, setbacks, and trauma (9). Prior research has demonstrated that resilience facilitates the development of positive coping strategies, enabling individuals to effectively address and overcome challenges and obstacles (10). In the nursing context, and particularly during periods of high occupational stress such as the COVID-19 pandemic, resilience has been shown to be critically important for sustaining nurses’ psychological wellbeing and enabling them to maintain professional and family role performances under extreme pressure (11). During the transition period, new graduate nurses often encounter transition shock; however, resilience functions as a protective mechanism that facilitates their ability to effectively manage and adapt to stressful circumstances, thereby mitigating the adverse impacts of stress (12). Notably, resilience not only buffers against immediate emotional distress but also supports the development of professional identity and sustained work engagement during periods of uncertainty and professional adjustment.

The notion of the psychological contract pertains to an individual’s beliefs regarding the reciprocal and implicit expectations existing between the individual and the organization (13). A breach of the psychological contract, consequently, denotes an employee’s perception that the organization has not satisfactorily fulfilled these expectations (14). Severl studies have demonstrated that breaches in psychological contracts adversely affect nurses’ work attitudes and engagement (15), perceived organizational citizenship (16), and turnover intention (17). However, the relationship between psychological contract and transition shock had not been explored.

In conclusion, recent studies mainly concentrated on the general magnitude of transition shock and its associated factors, ignoring the heterogeneity within the nursing population regarding transition shock. Consequently, this study seeks to (1) conduct a latent profile analysis to categorize the nursing population into distinct groups characterized by varying levels of transition shock, and (2) examine the predictors of transition shock among new graduate nurses. This approach aims to propose targeted interventions designed to mitigate transition shock among this demographic.

## 2 Methods

### 2.1 Design and participants

A convenience sampling, cross-sectional survey was conducted among new graduate nurses from September 2024 to October 2024 in a tertiary hospital in China. The inclusion criteria for the participants were listed as follows: (1) Currently employed nursing staff possessing a valid nursing practice certificate; (2) Employment duration of less than 1 year; (3) Ability to comprehend the questionnaire items and provide informed consent, thereby voluntarily participating in the research. The exclusion criteria are: (1) Participants with incomplete questionnaire responses; (2) Individuals who are currently on leave for further education or medical reasons. A total of 220 surveys were handed out, and 220 were successfully collected, achieving an effective rate of 100%.

Kendall’s sample size estimation technique recommends that the sample size be 5–10 times the number of independent variables. In this study, there were 12 independent variables, so the calculated sample size is at least 120. In addition, with an anticipated 20% of questionnaires being invalid, 144 patients are required for the survey. There were 220 valid questionnaires in this study. Consequently, the sample size gathered fulfilled the minimum criteria for the study.

### 2.2 Measures

#### 2.2.1 Sociodemographic characteristics

The sociodemographic variables examined in this research were gender, age, education, whether is only-child, family type, marital status, personality type, family residence relative to this hospital, prior internship experience at this hospital, and job satisfaction. Personality was assessed using a single self-report item asking participants to select which of three broad categories (Introverted, Moderate, or Extroverted) best described their usual behavioral style.

#### 2.2.2 Transition shock scale

Xue et al. (18) introduced the Transition Shock Scale of New Graduate Nurses in China, utilizing the Transition Shock Theory as its conceptual framework. This scale is specifically designed to assess the level of transition shock experienced by new graduate nurses. It comprises 27 items distributed across four dimensions: physical aspect (6 items), psychological aspect (8 items), sociocultural and developmental aspect (8 items), and knowledge and skills aspect (5 items). Participants were asked to evaluate each item using a 5-point Likert scale, ranging from 1 (totally disagree) to 5 (totally agree). The score for each dimension is determined by dividing the total score of that dimension by the number of items it includes. A higher score signifies a high level of transition shock. Specifically, scores exceeding 3.83 denote a high level of transition shock, scores between 2.17 and 3.83 indicate a moderate level, and scores below 2.17 reflect a low level of transition shock. The Cronbach’s alpha coefficient is 0.953.

#### 2.2.3 Connor-Davidson resilience scale

The CD-RISC, a self-report measure of psychological resilience, was developed by Connor and Davidson (19). Yu and Zhang (20) adapted and validated a Chinese version of this scale for a local population aged 18 years and older. The scale comprises 25 items that assess three dimensions: tenacity, strength, and optimism. Responses are recorded on a 5-point Likert scale ranging from 0 (not true at all) to 4 (almost always true). Due to its high reliability and validity, the scale is widely utilized in medical settings. The internal consistency coefficient of the Chinese version of the CD-RISC was reported to be 0.91. In the present study, the Cronbach’s alpha for this scale was found to be 0.956.

#### 2.2.4 Nurse psychological contract scale

The Nurse Responsibility Subscale of the Nurse Psychological Contract Scale, as developed by Chen (21), was employed to evaluate the extent to which nurses’ psychological contracts are fulfilled. This instrument encompasses three dimensions: developmental responsibility, team responsibility, and realistic responsibility, comprising a total of 17 items. A 5-point Likert scale was utilized, with response options ranging from “strongly agree” to “strongly disagree,” corresponding to scores from 1 to 5 for each item. The score for each dimension is determined by dividing the total score of that dimension by the number of items it includes. Higher total scores on the Psychological Contract Scale indicate a stronger perceived psychological contract breach. Lower scores indicate a more fulfilled psychological contract. Specifically, scores exceeding 4 denote a low level of psychological contract fulfillment, scores between 2 and 4 indicate a moderate level, and scores below 2 reflect a high level of psychological contract fulfillment. The Cronbach’s alpha coefficient is 0.968.

### 2.3 Data collection

Questionnaire Star, developed by Changsha Ranxing Information Technology Co., Ltd., served as the professional online platform for designing and linking the questionnaire. Prior to administering the questionnaire, participants were provided with standardized instructions detailing the content and purpose of the questionnaire, as well as important considerations for its completion. These instructions also underscored the principles of voluntary participation and the confidentiality of the information collected. Then, by clicking a link on their mobile devices, participants can access the online questionnaire and complete it. Each IP address was restricted to a single submission of the questionnaire to prevent multiple entries. After collecting the data, the questionnaire was checked to ensure it was complete, cleaned, and coded. Questionnaires were discarded if their response times were too short, under 90 s, or excessively long, over 600 s. The mean score rates were the actual average score divided by the highest score.

### 2.4 Data analysis

Statistical analyses were conducted with SPSS (version 26.0) and Mplus (version v8.7). For the descriptive statistics, continuous variables are presented as means and standard deviations, while categorical variables are reported as frequencies and percentages. To identify the latent profiles (subtypes) of transition shock among new graduate nurses, latent profile analysis (LPA) was conducted using the four dimensions of transition shock. LPA is a person-centered approach that identifies distinct subtypes based on heterogeneous characteristics within a sample. Individuals within each subtype share similar attributes, which aids in uncovering potentially diverse patterns of transition shock among new graduate nurse. The goodness-of-fit of the LPA models was evaluated using the following indices: the Akaike Information Criterion (AIC), the Bayesian Information Criterion (BIC), the sample-size-adjusted BIC (aBIC), the Lo–Mendell–Rubin (LMR) adjusted likelihood ratio test, the bootstrap likelihood ratio test (BLRT), and entropy. AIC, BIC, and ABIC assess model fit and parsimony. The LMR and BLRT compare the fit between the k − 1-class and k-class models, with lower *p*-values indicating that the k-class model fits better than the k − 1-class model. Entropy reflects the classification accuracy of the LPA model, with values closer to 1 indicating clearer classification. A well-fitting model is generally indicated by lower values of AIC, BIC, and ABIC; statistically significant *p*-values for the LMR and BLRT; and a higher entropy value (approaching 1) (22). Then, univariate and multivariate logistic regression analyses were employed to identify the factors associated with the different profiles derived from the LPA.

Specifically, one-way ANOVA and chi-square tests were used to examine the differences in demographic characteristics and each main variable across the different transition shock profiles. All variables with a *p*-value less than 0.05 in the univariate analysis were then selected as independent variables, with the transition shock profiles serving as the dependent variable, to construct a multinomial logistic regression model. This regression analysis was conducted to identify factors influencing the different transition shock profiles among new graduate nurses. In the initial multinomial logistic regression, the odds ratio for “not satisfied” versus “satisfied” was unrealistically large with an extremely wide confidence interval, indicating complete separation – all nurses with job dissatisfaction belonged to Class 2. To address this issue, we applied Firth’s bias-reduced penalized likelihood method (implemented via the brmultinom function in the R package brglm2 with type = “AS_mean”). Firth regression removes the first-order bias of maximum likelihood estimates and yields finite, consistent parameters even when separation occurs. The results presented (e.g., ORs, 95% CIs, and *p*-values) are based on this penalized approach. A *p*-value less than 0.05 was deemed statistically significant, while a *p*-value below 0.01 indicated a highly significant difference.

### 2.5 Ethical consideration

The study received approval from the Ethics Committee of the Central Hospital of Wuhan. The study followed the ethical guidelines, protocol, and regulations stated in the Declaration of Helsinki. Participants were thoroughly informed about the study’s objectives, and their informed consent was duly obtained. To safeguard participant privacy, access to all data was restricted exclusively to members of the research team.

## 3 Results

### 3.1 Participant characteristics

Among the participants, 10.9% were male, while 89.1% were female. Regarding age, 95.5% were younger than 25, while 4.5% were 25 or older. In terms of gender, 95% were Bachelor, while 5% were Master. Details were given in Table 1.

**Table 1:** Demographic characteristics and transition shock scores of investigated newly graduated nurses (*N* = 220).

Variable	*n* (%)	Class 1 (*n* = 29)	Class 2 (*n* = 145)	Class 3 (*n* = 46)	*x* ^2^	*p*
Gender						
Male	24 (10.9)	6	16	2	4.894	0.087
Female	196 (89.1)	23	129	44		
Age (years)						
≥25	10 (4.5)	1	7	2	0.111	0.946
<25	210 (95.5)	28	138	44		
Education						
Bachelor	209 (95.0)	28	137	44	0.27	0.874
Master	11 (5.0)	1	8	2		
Whether is only-child						
Yes	61 (27.7)	10	40	11	0.996	0.608
No	159 (72.3)	19	105	35		
Family type						
Single-parent family	25 (11.4)	2	19	4	3.861	0.425
Two-parent family	187 (85.0)	26	119	42		
Restructured family	8 (3.6)	1	7	0		
Marital status						
Single	219 (99.5)	29	145	45	3.800	0.150
Married	1 (0.5)	0	0	1		
Personality						
Introverted	51 (23.2)	3	32	16	15.647	0.004**
Moderate	145 (65.9)	18	98	29		
Extroverted	24 (10.9)	8	15	1		
Family residence relative to this hospital						
Yes	30 (13.6)	6	18	6	1.423	0.491
No	190 (86.4)	23	127	40		
Prior internship experience at this hospital						
Yes	39 (17.7)	4	25	10	0.839	0.657
No	181 (82.3)	25	120	36		
Job satisfaction						
Not satisfied	9 (4.1)	0	7	2	23.891	<0.001***
Neutral	95 (43.2)	3	63	29		
Satisfied	116 (52.7)	26	75	15		

**p < 0.01; ***p < 0.001.

### 3.2 The items and dimensions scores associated with transition shock of investigated new graduate nurses

The mean score of transition shock in this study was 2.97. Among the various dimensions of transition shock, the highest dimension is physical while the lowest dimension is social culture and development, as shown in Table 2.

**Table 2:** The items and dimensions scores associated with transition shock of investigated newly graduated nurses.

Variables	*n*	Min	Max	Mean	Mean score rates
Transition shock (total)	220	1	4.96	2.97	59.37%
Physical	220	1	5	3.19	63.70%
Psychological	220	1	5	2.86	57.28%
Knowledge and skills	220	1	5	3.07	61.40%
Social culture and development	220	1	5	2.85	56.93%

The five items receiving the highest scores concerning challenges faced by new graduate nurses transitioning into their roles are as follows: “Too tired to do anything after work”, “I feel very anxious when encountering unfamiliar operations”, “Sometimes there are bloodshot eyes or dark circles under the eyes”, “Sometimes I have to work overtime to get the job done” and “Always feel sleepy”. The mean scores rates for these items were 69.4, 67.4, 66.0, 63.4, and 62.6%, respectively (refer to Table 3). Notably, three of these items pertain to the physical dimension. These findings indicate that physical exhaustion and fatigue are the most prominent challenges among new graduate nurses during transition, suggesting that workplace support strategies should prioritize mitigating physical burden.

**Table 3:** Mean score rates of transition shock items among newly graduated nurses (Top Five).

The items	Mean	Min	Max	Mean score rates	The dimension to which it belongs to
Too tired to do anything after work.	3.47	1	5	69.4%	Physical
I feel very anxious when encountering unfamiliar operations.	3.37	1	5	67.4%	Psychological
Sometimes there are bloodshot eyes or dark circles under the eyes.	3.30	1	5	66.0%	Physical
Sometimes I have to work overtime to get the job done.	3.17	1	5	63.4%	Knowledge and skills
Always feel sleepy.	3.13	1	5	62.6%	Physical

### 3.3 LPA of transition shock

As illustrated in Table 4, we examined between one and six potential profile models. The AIC, BIC, and aBIC values consistently decreased with an increase in the number of latent profiles. Nevertheless, the *p*-values for the LMR tests of the four-class and five-class profile models were not statistically significant, suggesting that these models did not offer a statistically significant improvement over the three-class profile model. According to the model fit indices, Model 3 had the best fit.

**Table 4:** Latent class model fit comparison.

Model	K	AIC	BIC	aBIC	Entropy	P_LMRT_	P_BLRT_	Probability of class
1	8	1,993.186	2,020.335	1,994.983				
2	13	1,759.403	1,803.52	1,762.323	0.795	0.084	<0.001	0.277/0.723
3	18	1,555.844	1,616.929	1,559.887	0.901	0.004	<0.001	0.132/0.659/0.209
4	23	1,510.077	1,588.13	1,515.243	0.857	0.159	<0.001	0.055/0.545/0.214/0.186
5	28	1,486.994	1,582.016	1,493.284	0.873	0.318	<0.001	0.059/0.200/0.541/0.182/0.018
6	33	1,475.244	1,587.234	1,482.657	0.890	0.278	<0.001	0.059/0.200/0.191/0.527/0.018/0.005

AIC, Akaike Information Criterion; BIC, Bayesian Information Criterion; aBIC, adjusted Bayesian Information Criterion; LMR (P), *p* value for the Lo–Mendell–Rubin; BLRT (P), *p* value for the Bootstrapped Likelihood Ratio Test.

**Figure 1:**
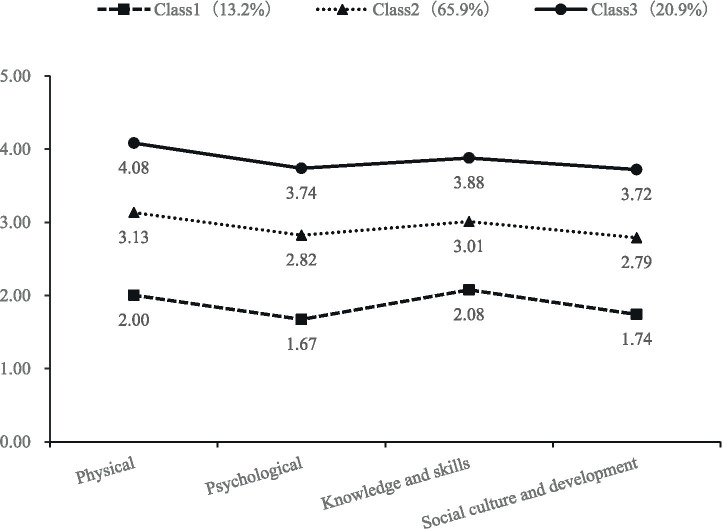
Three subtypes of transition shock among new graduate nurses based on the latent profile analysis.

Figure 1 depicts the three distinct profiles identified by the latent profile analysis (LPA). These profiles were labeled as follows: low transition shock (13.2%, Class 1), middle transition shock (65.9%, Class 2), and high transition shock (20.9%, Class 3).

### 3.4 Univariate analysis of transition shock

Univariate analysis was employed to evaluate the data gathered. According to Tables 1, 5, significant differences in transition shock were observed in variables such as personality, job satisfaction, resilience, and psychological contract (*p* < 0.05).

**Table 5:** Differences in scores of resilience and psychological contract across different latent classes.

Item	Total scores	Class 1 Low transition shock	Class 2 Middle transition shock	Class 3 High transition shock	*F*	*p*	Multiple comparisons
Resilience	60.92 ± 15.81	73.86 ± 16.47	58.64 ± 13.85^a^	59.96 ± 17.57^a^	12.491	<0.001	Class 1 < Class 2 Class 1 < Class 3
Psychological contract	1.94 ± 0.62	1.43 ± 0.46	1.99 ± 0.59 ^a^	2.13 ± 0.65^a^	14.037	<0.001	Class 1 < Class 2 Class 1 < Class 3

Data are Mean ± SD. ^a^Compared with the low transition shock group, *p* < 0.05. Higher scores on the Psychological Contract Scale indicate more severe breach (worse fulfillment).

### 3.5 Multivariate analysis of transition shock

A multinomial logistic regression analysis was conducted with the three categories of transition shock in new graduate nurses as the dependent variable, using statistically significant factors from the prior univariate analysis as independent variables. The findings, as presented in Table 6, indicated that personality, job satisfaction, resilience, and psychological contract were significant predictors of transition shock among new graduate nurses.

**Table 6:** Multinomial logistic regression analysis of the latent categories of transition shock among new graduate nurses.

Variables	*β*	SE	OR	95%CI	*p*
Class 2 (ref.: Class 1)					
Resilience	−0.035	0.017	0.97	0.93–0.99	0.041*
Psychological contract	1.023	0.483	2.78	1.08–7.16	0.034*
Class 3 (ref.: Class 1)					
Psychological contract	1.485	0.554	4.42	1.49–13.08	0.007**
Class 2 (ref.: Class 3)					
Job satisfaction (ref.: Satisfied)					
Neutral	−0.847	0.395	0.43	0.20–0.93	0.032*
Personality (ref.: Extroverted)					
Introverted	−2.418	1.120	0.09	0.01–0.80	0.031*
Resilience	−0.027	0.013	0.97	0.95–0.99	0.039*

Class 1 = low transition shock; Class 2 = middle transition shock; Class 3 = high transition shock. Higher scores on the Psychological Contract Scale indicate more severe breach (worse fulfillment). *p < 0.05; **p < 0.01.

The findings suggest that, in comparing the middle transition shock group with the low transition shock group, new graduate nurses experiencing a significant breach of psychological contract were more frequently categorized within the middle transition shock group. Conversely, those exhibiting high psychological resilience were more likely to be classified into the low transition shock group. In the comparison between the high transition shock group and the low transition shock group, nurses with a substantial psychological contract breach were predominantly assigned to the high transition shock group. Furthermore, when contrasting the middle transition shock group with the high transition shock group, nurses characterized by neutral job satisfaction, an introverted personality, and high psychological resilience were more inclined to be associated with the high transition shock group.

## 4 Discussion

### 4.1 The latent profile of transition shock among new graduate nurses

This study identified three distinct latent profiles of transition shock among new graduate nurses: low, middle and high transition shock groups, thereby highlighting significant heterogeneity among individuals.

In the study, 20.9% of the surveyed nurses were classified within the high transition shock group, while 65.9% were categorized under the moderate transition shock group. Within this specific hospital, a notable proportion of new graduate nurses experienced a relatively high level of transition shock, which is broadly consistent with findings from similar studies (12, 23). However, given the single center design, the exact percentage distributions should be interpreted as exploratory and institution-specific; multi-center validation is needed before these figures can be used as reference benchmarks for other populations. Notably, nurses in the high transition shock group demonstrated elevated scores across all four assessed dimensions, highlighting substantial challenges in physical, psychological, knowledge and skills, as well as socio-cultural and developmental domains during their transition. The physical dimension, in particular, exhibited the highest scores, indicating that new graduate nurses may experience considerable physical discomfort as they transition from student roles to practicing professionals. Several factors may be associated with this finding: (1) Role Transition and Increased Work Intensity: Despite the completion of clinical internships prior to graduation, the transition from a student to a professional nurse involves substantial alterations in the nature of work tasks, the intensity of responsibilities, and the level of pressure experienced (24). The frequent physical demands intrinsic to clinical nursing, such as patient lifting and transferring, prolonged periods of standing, and night shift rotations, are likely linked to significant physical fatigue (25). This fatigue has been reported as the primary and most immediately perceived and reported stressor among new graduate nurses. (2) Unfamiliarity with Skills Exacerbates Psychosomatic stress: Insufficient proficiency in operational techniques not only diminishes work efficiency but also may predispose individuals to muscular tension and awkward movements during procedures (26). Concurrently, the sustained state of heightened mental vigilance required to ensure patient safety continuously depletes psychological resources, which may be associated with somatization symptoms such as headaches and insomnia (27). This suggests that managers should pay close attention to the physical wellbeing of new graduate nurses by implementing practical measures such as reasonable flexible scheduling to reduce overtime demands and establishing designated rest days to ensure adequate recovery. These actions could help address the high levels of physical fatigue observed in this study, thereby supporting a healthier and more sustainable transition into clinical practice.

### 4.2 Factors predicting transition shock among new graduate nurses

#### 4.2.1 Job satisfaction

This study finds that, among new graduate nurses, those with neutral (moderate) job satisfaction were significantly less likely to belong to Class 2 (a middle transition shock profile) compared with Class 3 (a high transition shock profile). However, dissatisfaction was not a statistically significant predictor of class membership in any comparison after Firth penalization, contrary to our initial expectation. The result is not agreement with findings from related research (28). One possible explanation is that the extreme group of “dissatisfied” nurses was very small (*n* = 9), which limited statistical power despite the use of bias-reduced estimation. Alternatively, moderate satisfaction might serve as a buffer against the most severe transition shock, whereas complete dissatisfaction may be so rare or so strongly confounded with other factors (e.g., low resilience, poor psychological contract) that its independent effect becomes negligible. Future studies with larger samples are needed to clarify the role of extreme dissatisfaction.

#### 4.2.2 Personality

This study provides evidence that an introverted personality is an independent and significant factor influencing the experience of transition shock among new graduate nurses. The analysis reasons are as follows. Within the framework of Chinese culture, introverted novice nurses tend to process information internally, may render them more vulnerable to psychological and cognitive stress in high-pressure clinical environments characterized by significant interpersonal demands (29). Influenced by collectivist and relationship-oriented cultural values, these nurses often prefer engaging in deep communication within small groups, which may marginalize them within medical teams that emphasize harmony and interpersonal connections. Consequently, they may encounter challenges in accessing timely emotional support and practical guidance (30). Additionally, traditional Chinese virtues that prioritize humility, restraint, and the avoidance of drawing attention can deter them from actively seeking assistance or expressing uncertainties (30). As a result, their learning needs may be neglected, and errors may not be promptly addressed, which may exacerbate the practice competence gap. Furthermore, introversion is closely associated with concerns about face preservation and sensitivity to negative evaluation, making these nurses particularly reactive to feedback from superiors and peers. This increased vigilance, fundamentally anchored in the apprehension of social evaluation, may exacerbate their experience of occupational stress. It is crucial to emphasize that introversion should not be regarded as a deficiency. The issue stems from the tendency of many modern nursing work environments to implicitly favor extroverted, proactive behavioral models. This potential misalignment between individual characteristics and environmental expectations poses unique adaptation challenges for new practicing introverted nurses. It is crucial for nurse managers to actively recognize and address the particular needs of introverted nurses, who may encounter significant adaptation pressures yet remain reticent about their challenges. Fostering an inclusive environment is vital for the successful integration of these individuals (28); this can be achieved through the establishment of secure communication channels, strategic mentorship pairings, and the promotion of a culture that minimizes obstacles to seeking support.

#### 4.2.3 Resilience

In this study, a comparison between new graduate nurses experiencing high and low levels of transition shock revealed that those with strong psychological resilience were more frequently categorized into the low transition shock group. However, nurses with high resilience were also observed in the high transition shock group. Psychological resilience, as an intrinsic trait, is thought to serve a protective function by enabling individuals to withstand external stressors and internal psychological challenges (9). The finding that some resilient nurses still experienced high transition shock does not negate the protective role of resilience. Instead, it suggests that resilience alone may be insufficient to buffer against extremely adverse work environments—for example, when accompanied by severe psychological contract breach, persistent excessive workload, or inadequate organizational support. In such contexts, even individuals with high resilience may remain vulnerable to high levels of transition shock. Therefore, while fostering resilience remains important, managers should also address modifiable environmental risk factors, such as fulfilling psychological contracts, reducing excessive physical burden, and providing adequate social support, to more effectively prevent high transition shock among new graduate nurses.

#### 4.2.4 Psychological contract

The findings of this study suggest that the extent of an unfulfilled psychological contract is significantly associated with transition shock among new graduate nurses. Specifically, a higher incidence of psychological contract breach correlates with an increased experience of transition shock. This relationship may be partly explained by the negative correlation identified between psychological contract breach and work engagement within the nursing profession (31). When the actual support, development opportunities, work environment, and other conditions provided by the hospital or nursing team substantially diverge from the expectations established by new nurses based on recruitment promises and social cues, there is a tendency for these nurses to exhibit diminished levels of work engagement. Consequently, they may encounter heightened challenges and pressures related to role adaptation, skill application, interpersonal relationships, and both physiological and psychological wellbeing. This escalation may contribute to an increased level of transition shock and intensifies their intention to leave the profession (32). This underscores the necessity for managers to focus on fulfilling the psychological contracts of new graduate nurses. To address this issue, strategies such as capitalizing on their strengths, cultivating a positive organizational climate, and offering career planning guidance should be implemented.

### 4.3 Limitations and future studies design

While this study offers empirical insights into the factors affecting the impact of transition shock on new graduated nurses, this study has limitations in the following areas. Firstly, the sample size is relatively limited and is derived from a single medical institution, potentially restricting the representativeness of the sample. Future research could enhance validity through multi-center, large-sample stratified sampling. Secondly, the personality was measured by a simple self-reported question rather than a validated instrument, which may have introduced measurement bias and limited our ability to capture the multifaceted nature of personality. Future studies should adopt standardized tools like the Big Five Inventory to assess personality more reliably. Thirdly, the cross-sectional design poses challenges in establishing causal temporal relationships between variables. Lastly, the identified profiles may primarily reflect severity differences rather than fully distinct qualitative subgroups. In light of these considerations, future research could focus on conducting multi-center, large-sample longitudinal studies to dynamically observe the evolution of transformation shocks and their interactions with influencing factors, such as personality traits, job satisfaction, resilience, and psychological contract.

## Data Availability

The raw data supporting the conclusions of this article will be made available by the authors, without undue reservation.
